# Effects of Tea Consumption on Anthropometric Parameters, Metabolic Indexes and Hormone Levels of Women with Polycystic Ovarian Syndrome: A Systematic Review and Meta-Analysis of Randomized Controlled Trials

**DOI:** 10.3389/fendo.2021.736867

**Published:** 2021-12-13

**Authors:** Wenjuan Shen, Yujia Pan, Bao Jin, Zongyu Zhang, Tianjiao You, Yangfan Qu, Mei Han, Xingxing Yuan, Yang Zhang

**Affiliations:** ^1^ Department of Obstetrics and Gynecology, First Affiliated Hospital of Heilongjiang University of Chinese Medicine, Harbin, China; ^2^ Department of Obstetrics and Gynecology, Heilongjiang University of Chinese Medicine, Harbin, China; ^3^ Centre for Evidence-Based Chinese Medicine, Beijing University of Traditional Chinese Medicine, Beijing, China; ^4^ Department of Gastroenterology, Heilongjiang Academy of Traditional Chinese Medicine, Harbin, China; ^5^ Department of Internal Medicine, First Affiliated Hospital of Heilongjiang University of Chinese Medicine, Harbin, China

**Keywords:** tea, polycystic ovary syndrome, meta-analysis, systematic review, complementary therapy

## Abstract

**Objective:**

Our aim was to conduct a systematic review and meta-analysis to assess the effectiveness and safety of tea supplements for patients with polycystic ovary syndrome (PCOS).

**Methods:**

We conducted searches of the published literature in PubMed, EMBASE, Cochrane Library, Web of Science, Chinese Biomedical Literature Database, Chinese National Knowledge Infrastructure (CNKI), VIP database, and Wanfang Database in 1985 to September 2021. Data from randomized controlled trials (RCTs) were obtained to assess the effects of tea versus placebo in women with PCOS. Weighted mean differences (WMDs) were pooled using a random-effects model or risks ratios (RRs) using a random-effects model.

**Results:**

Six RCTs (235 participants) were included in our systematic review. Tea supplements as adjuvant therapy led to greater improvement in body weight (WMD −2.71, 95% CI −4.95 to −0.46, *P* = 0.02, I^2^ = 0%), fasting blood glucose (FBG: WMD −0.40, 95% CI −0.59 to −0.20, *P* < 0.0001, I^2^ = 0%) and fasting insulin (FINS: WMD −3.40, 95% CI −4.76 to −2.03, *P* < 0.00001, I^2^ = 0%) when compared with placebo. There were no significant differences of body mass index, waist circumference, hip circumference, waist-to-hip ratio (WHR), body fat rate, total testosterone, free testosterone (FT), dehydroepiandrosterone, luteinizing hormone or follicular-stimulating hormone (FSH) between the two groups. In addition, subgroup analysis suggested that green tea was effective on body weight, FINS, FBG, FT, and FSH, and herbal tea can also reduce FT levels, tea supplements had a significant impact on FBG and FSH in trials with intervention duration ≥ 3 months, and intervention lasting less than 3 months can improve FINS. Tea had significant effect on reducing WHR, FBG and FSH in Asian PCOS patients, but not in Caucasians. And there was no statistically significant effect of tea on weight and FINS in Asians, but it was effective for Caucasian participants. Compared with placebo, tea supplements did not cause significant adverse reactions (RR 1.45, 95% CI 0.30 to 6.90, *P* = 0.65, I^2^ = 0%).

**Conclusion:**

This meta-analysis suggests that consumption of tea supplementation in women with PCOS could significantly decrease the levels of FBG and FINS as well as reduce body weight. Especially green tea, not only has the above effects, but also has a significant effect on improving a variety of reproductive hormone indexes. Furthermore, tea supplementation is a relatively safe therapy for PCOS patients.

**Systematic Review Registration:**

PROSPERO https://www.crd.york.ac.uk/PROSPERO/display_record.php?RecordID=212755, identifier CRD42021249196.

## Background

Polycystic ovary syndrome (PCOS) is a common reproductive endocrine disease characterized by menstrual disorders, oligo-ovulation/anovulation, hyperandrogenism, polycystic ovaries, and insulin resistance (IR), and it affects 18–22% of women (1). PCOS can also lead to infertility and miscarriage, it not only increases the health burden of the affected women, but also greatly reduces their quality of life. In the US alone, PCOS patients spend more than $4 billion on the treatment and care of the disease, which puts a tremendous economic burden on patients (2). However, the etiology and pathogenesis of PCOS are complex, there is no curative treatment for PCOS at present.

Some studies have shown that obesity is an important factor in the occurrence of PCOS, and reducing body weight is an effective method for improving the clinical symptoms of PCOS (3–5). It has been proposed in the American Society for Reproductive Medicine 2018 Guidelines that lifestyle interventions including diet therapy should be used as the first-line treatment of PCOS. Dietary interventions can not only improve obesity, hyperandrogenemia, IR, and other clinical manifestations, but can also reduce the occurrence of a variety of complications including type 2 diabetes mellites, atherosclerosis, cardiovascular disease, and so on. Thus, it can be seen that dietary interventions have positive effects in the treatment of PCOS (6, 7).

Tea, derived from the fresh leaves of *Camellia sinensis*, is a common beverage consumed daily in many countries (8). Diet is regarded as one of the important means of complementary and alternative medical therapy, and tea plays an important role in diet and medicine (9, 10). With the development of the tea industry, tea culture has risen rapidly, and tea has been gradually accepted by people around the world (11). Nowadays, many people are interested in its efficacy in preventing various diseases (12). Tea has high nutritional value and contains more than 20 elements needed by the human body, and it has many functions such as stimulating the central nervous system, improving immunity, anti-oxidation, and regulating glucose and lipid metabolism disorder (11). In addition, it has been shown that tea and tea extracts have beneficial effects on body weight, body fat rate (BFR), glucose, insulin, and free testosterone (FT) in patients with PCOS (13, 14).

There are many kinds of tea, including green tea (unfermented), black tea (fully fermented), and oolong tea (semi-fermented) according to different fermentation forms, as well as some herbal teas (15–18). Green tea is the most productive of all teas and is rich in catechins, and epigallocatechin gallate (EGCG) is the most abundant green tea catechin. It has been confirmed that EGCG can inhibit adipocyte differentiation and proliferation leading to weight loss in cultured adipocyte models (19). In animal models of obesity, EGCG was found to promote beta-oxidation in mice and to enhance energy expenditure (20). In addition, according to Liu et al.’s (21) meta-analysis green tea has a good effect on reducing FBG, glycated hemoglobin (HbA1c) concentrations, and FINS. Western medicine treatments often have some adverse reactions and high costs, and it has been reported that more and more PCOS patients are unsatisfied with oral western medicine, such as metformin, orlistat, and Diane-35 as treatments for PCOS, and they express a strong receptiveness for seeking supplementary and alternative treatments (22). Various types of tea can cater to the different taste preferences of PCOS patients and provide more choices for them. Tea supplements have the advantages of convenience, limited adverse reactions, and high acceptance, so they have great potential for popularization.

However, the results of some studies investigating the effects of tea supplements in PCOS patients have been inconsistent (14, 17, 18, 23). Currently, there is a lack of evidence for using tea supplements to treat patients with PCOS. Therefore, we performed a systematic review and meta-analysis of existing studies in order to evaluate the efficacy and safety of tea supplements in the treatment of PCOS and thus provide a reliable basis for clinical practice.

## Protocol and Registration

This systematic review and meta-analysis was designed and conducted in accordance with a predetermined protocol according to the Cochrane Handbook’s recommendations (24). We reported the results according to the Preferred Reporting Items for Systematic Reviews and Meta-Analyses (PRISMA) (25). The prospective review has been registered in PROSPERO: CRD42021249196. https://www.crd.york.ac.uk/PROSPERO/display_record.php?RecordID=249196.

### Literature Search Strategy

Studies were identified by searching electronic bibliographic databases including PubMed, EMBASE, Cochrane Library, Web of Science, Chinese Biomedical Literature Database (CBM), Chinese National knowledge Infrastructure (CNKI), the VIP database, and the Wanfang Database. No limits were applied for language. We used controlled vocabulary (MeSH in PubMed and Emtree in Embase) and keywords as search terms. The last search was run on September 8, 2021. The full details of search strategy are available (***Supplementary Appendix 1***). At the same time, we manually searched the reference lists of identified papers and the grey literature to further look for studies that might meet the selection criteria.

### Eligibility Criteria

We carried out the initial search, deleted duplicate records, screened the titles and abstracts for relevance, and identified records as included, excluded or uncertain. In case of uncertainty, the full-text article was acquired to identify eligibility. The eligibility criteria were according to the PICOS format (26) and included the following: participants (P), intervention (I), control (C), outcome (O), and study design (S). Two researchers were responsible for independently evaluating the identified articles (**Table 1**). If disagreement arose, a third researcher would make the judgment.

**Table 1:** Eligibility criteria.

Inclusion criteria	
Participants	Patients with a diagnosis of PCOS
Interventions	The intervention group was given tea supplements (not limited by dosage, dosage form, frequency, or duration).
Comparisons	The control group was treated with blank or placebo.
Outcomes	The primary outcome: Body weight. The secondary outcomes (1): Clinical results: BMI, WC, HC, WHR, and BFR (2); Metabolic results: FBG and FINS (3); Hormone parameters: total testosterone (TT), FT, DHEAS, luteinizing hormone (LH), and follicular-stimulating hormone (FSH).
Study type	Randomized controlled trials (RCTs) assessing the effects of tea supplements on PCOS.

### Data Extraction

Data extraction was performed by two researchers independently who thoroughly reviewed each included article. Any disagreements in the data extraction were resolved by discussion to reach a consensus in all cases. Data collected from each study including the following: basic information of the articles (author, country, and publication year) and participants (race, mean age, mean weight, and sample size), details of tea supplements (species, dosage form, dosage, and treatment duration), comparison methods, every outcome parameter, and adverse reactions. We strived to ensure the integrity of the data, and missing data were requested by contacting the authors *via* email or telephone.

### Risk-of-Bias Assessment

We used the Cochrane Collaboration risk-of-bias instrument for RCTs to conduct the quality assessment (24). Factors related to bias risk included random sequence generation, allocation concealment, application of blinding method, data integrity, selective reporting with or without results, and other sources of bias. Every domain was answered “yes,” “no,” or “unclear.” We reached risk-of-bias judgements according to the standards of The Cochrane Collaboration and assigned three categories – unclear, low, and high risk of bias. The analysis was independently completed and cross-checked by two researchers, and any divergence was resolved by discussion with a third researcher.

### Statistical Analysis

The meta-analyses were performed by computing weighted mean difference (WMD) or risks ratios (RRs) with 95% confidence intervals (CIs) using a random-effects model, accounting for clinical heterogeneity. Heterogeneity across studies was assessed by using the Q statistic with its p value and *I^2^* statistic (23). The *I^2^* statistics used to quantify the proportion of total variation in the effect estimation that is due to between study variations. An *I^2^* value greater than 50% indicates significant heterogeneity (18). A two-sided p value less than 0.05 was considered statistically significant. All analyses were performed using Stata statistical software version 15.0 (StataCorp, USA).

## Results

### Study Selection

**Figure 1:**
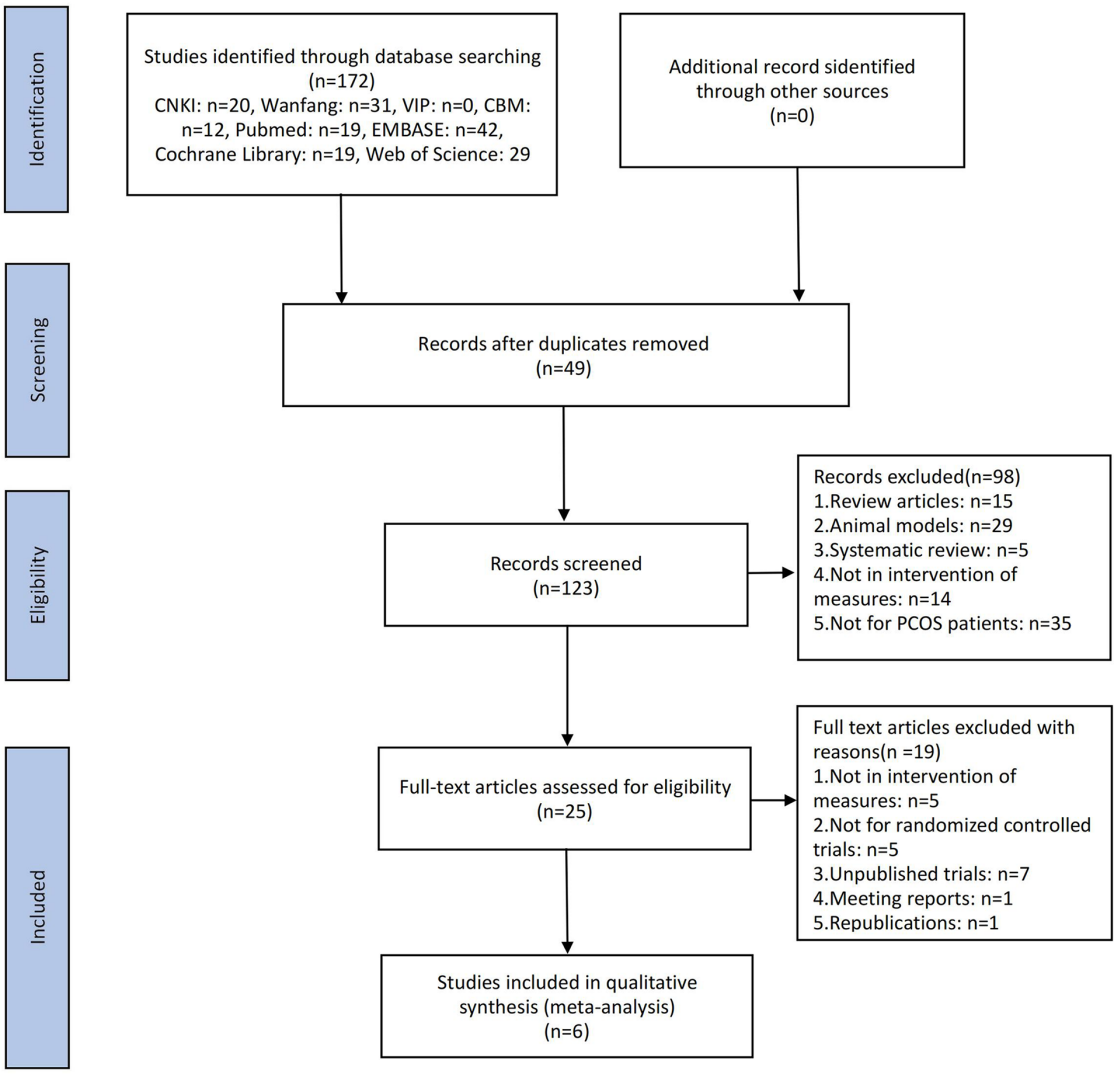
Flow diagram of the study selection process.

Initially, 172 articles were identified in the database searches, including 19 articles from PubMed, 42 articles from EMBASE, 19 articles from Cochrane Library, 29 articles from Web of Science, 12 articles from CBM, 20 articles from CNKI, and 31 articles from Wanfang Database (**Figure 1**). A total of six publications including 235 patients with PCOS were included in the meta-analysis after screening the titles, abstracts, and full texts (14, 17, 18, 23, 27, 28).

### Characteristics of the Studies

All of the studies were RCTs and were published in English. The studies were conducted in the following areas: China (23), Iran (14, 27, 29), Britain (17), and Jordan (18). Four studies reported the details of the method of random assignment using either computer-generated numbers, block randomization methods, or a table of random numbers (17, 18, 23, 28), and three trials mentioned specific double-blind methods (18, 23, 27). All participants were diagnosed with PCOS according to guidelines set by the Rotterdam criteria, and they ranged in age from 18 to 42 years old. In four studies, all patients had the characteristics of obesity or overweight (14, 23, 27, 28). Of the total sample in one trial, 64% comprised overweight or obese individuals and 20% had IR (18). Participants in the remaining one trial all had hirsutism (17). Two articles noted that none of the participants took medication due to infertility during the observation periods (14, 28). Among the six included RCTs, two of them administered the intervention to participants in two cups of tea per day made from herbal tea bags (17, 18), two of them in capsules (14, 23), and two in tablets with daily doses of 500mg (27, 28). The duration of the intervention varied from 1 to 3 months. Three tea supplements were investigated in the included studies, green tea was investigated in four studies (14, 23, 27, 28), one study investigated marjoram tea (18), and one study investigated spearmint herbal tea (17). In 5 of the 6 studies, tea supplements were compared with placebo (14, 17, 18, 23, 27), while one study compared tea supplements with placebo and metformin respectively (28). In two studies, all participants were given dietary advice and instructed not to consume any beverages or food that contain caffeine or polyphenols (23, 27). In two trials, the patients were contacted periodically to ensure medication compliance (14, 23). The participants were assessed in all trials at baseline and immediately at the conclusion of the study period, and two trials conducted additional mid-term assessments (17, 28). Five trials investigated the effect of tea supplements on weight (14, 18, 23, 27, 28), and three reported on BMI and WHR (23, 27, 28). Three studies reported on reproductive hormone indexes, such as TT, FSH, LH, and DHEAS (17, 18, 23). Three studies analyzed the effect of tea on FINS (14, 18, 23), two of which measured the level of FBG (18, 23). Only one study reported on the outcomes of the dermatology quality of life index (DLQI) and Ferriman-Gallwey score (17) (**Table 2**).

**Table 2:** The characteristics of the included studies.

Author, year	Nation	Sample size (T/C)	Mean age (T/C)	Weight (T/C)	Intervention	Comparison	Dosage form	Dosage	Treatmentduration	Outcome measured	Adverse reaction
Tehrani et al., 2017 (14)	Iran	30/30	20–40	86.68 ± 6.86/86.28 ± 6.03	Green tea	Placebo	Capsule	500 mg	12 weeks	Weight, FINS, FT	Not reported
Grant, 2009 (17)	Britain	21/20	25.5 (19–42)	Not reported	Spearmint herbal tea	Placebo	Herbal tea bags	2 cups	30 days	FT, TT, DHEAS, LH, FSH, DLQI, FG	None
Husein et al., 2015 (18)	Jordan	14/11	21.1 ± 1.2/21.5 ± 1.0	66.04 ± 2.84/67.02 ± 2.87	Marjoram tea	Placebo tea	Herbal tea bags	2 cups (250 ml each)	1 month	Weight, FSH, LH, E2, P, TT, DHEAS, FINS, FBG, HOMA-IR, GIR	T: bloating (n = 1), nausea (n = 1), mild sedation (n = 1), more frequent urination (n = 5) C: bloating (n = 1) more frequent urination (n = 2)
Chan et al., 2006 (23)	China	18/16	34.8 ± 4.2/30.9 ± 2.4	76.0 (70.3–80.6)/76.6 (68.2–79.7)	Green tea	Placebo	Capsule	6 capsules (contained an equivalent of 540 mg EGCG)	3 months	Weight, BMI, WHR, BFR, TT, FAI, A2, DHEAS, FSH, LH, SHBG, FINS, FBG, GIR, FLP, FC, TG, LDL, HDL, non-HDL-C	T: none C: gastrointestinal discomfort (n = 1)
Mombaini et al., 2017 (27)	Iran	22/23	23.22 ± 5.24/24.17 ± 6.83	73.99 ± 21.53/71.40 ± 12.27	Green tea	Placebo	Tablet	500 mg	45 days	Weight, BMI, WC, HC, WHR, BFR, IL-6, hsCRP, TNF-α	T: gastrointestinal complications (n = 2) C: none
Farhadian et al., 2020 (28)	Iran	15/15	18–35	75.33 ± 6.47/74.46 ± 7.59/	Green tea	Placebo	Tablet	500 mg	3 months	Weight, BMI, WC, HC, WHR	Not reported

BMI, body mass index; WC, waist circumference; HC, hip circumference; WHR, waist-to-hip ratio; BFR, body fat rate; TT, total testosterone; FT, free testosterone; DHEAS, dehydroepiandrosterone sulfate; A2, androstenedione; FAI, free androgen index; FSH, follicle-stimulating hormone; LH, luteinizing hormone; E2, estradiol; P, progesterone; FINS, fasting insulin; HOMA-IR, homeostasis model assessment of insulin resistance; SHBG, sex hormone-binding globulin; FBG, fasting blood glucose; GIR, fasting blood glucose/fasting insulin ratio; FLP, fasting leptin; FC, fasting cholesterol; TG, triglycerides; LDL, low-density lipoprotein; HDL, high-density lipoprotein; non-HDL-C, fasting non-HDL cholesterol; IL-6, interleukin 6; hsCRP, high-sensitivity C-reactive protein; TNF-α, tumor necrosis factor-α; DLQI, dermatology quality of life index; FG, Ferriman-Gallwey score.

### Risk-of-Bias Assessment

**Figure 2:**
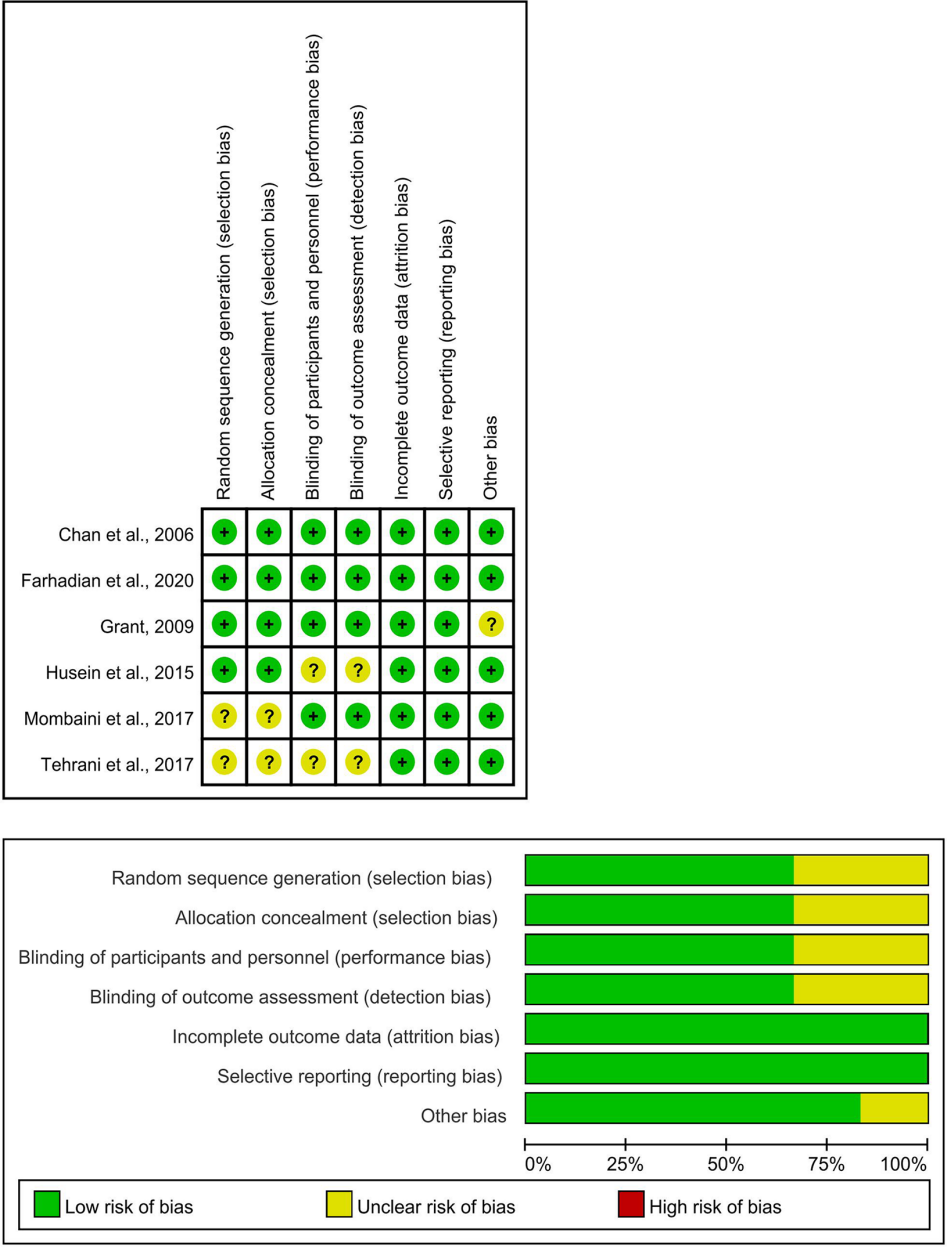
Risk of bias.

The risk of bias related to random sequence generation and allocation concealment in most included RCTs was low. Two trials were assessed to have an unclear risk of selection bias due to have no randomization and allocation details provided (14, 27). Reports of double-blind methods and allocation concealment were insufficient in two studies (14, 18), which were judged to have unclear risk for detection bias. One study was assessed as having unclear risk for other biases because of lack of description of the baseline (17), while the baseline characteristics of the other five trials were balanced (14, 18, 23, 27, 28), judged them as having a low risk of bias. There was no bias for selective reporting in any of the studies (**Figure 2**). The specific evaluation basis is available (***Supplementary Appendix 2***).

## Outcome Measures

### Effect of Tea Supplements on Anthropometric Parameters

**Figure 3:**
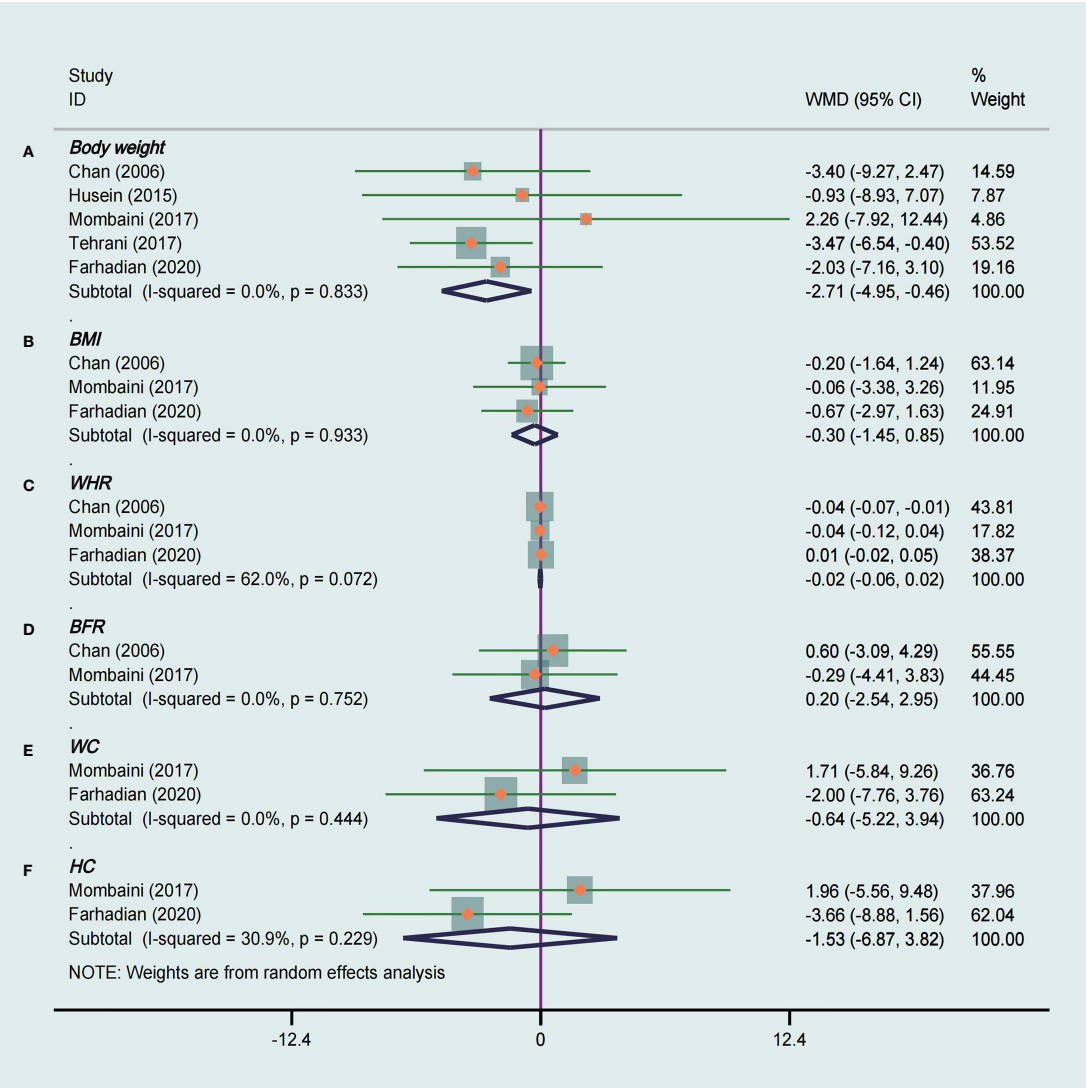
Meta-analyses of the effect of tea supplements on anthropometric parameters. **(A)** Body weight, **(B)** BMI, **(C)** WHR, **(D)** BFR, **(E)** WC, and **(F)** HC.

Five studies (14, 18, 23, 27, 28) analyzed of the effects of tea supplements on weight in 194 patients. Compared with the placebo group, there was a significant decrease in body weight in the tea supplement group (WMD −2.71, 95% CI −4.95 to −0.46, *P* = 0.02, I^2^ = 0%, **Figure 3A**). In addition, a subgroup analysis was performed in view of the fact that different types of tea, duration of intervention and races of participants may have different effects on body weight. The stratified analysis revealed that green tea had a significant effect on weight loss (WMD −2.86, 95% CI −5.19 to −0.52, *P* = 0.02, I^2^ = 0%), while herbal tea did not (**Table 3**). At the same time, it was observed that tea supplements had weight loss effect on Caucasian PCOS patients (WMD −2.59, 95% CI −5.01 to −0.16, *P* = 0.04, I^2^ = 0%), but not in Asian patients (**Table 3**).

**Table 3:** Subgroup analyses of the effect of different tea types or intervention duration in PCOS.

Outcome	Feature	Subgroups	Studies	Participants	Effect estimate WMD 95% CI	P Value	I^2^ (%)
Weight	Tea type	Green tea	4	169	–2.86 [–5.19, –0.52]	*P* = 0.02	I^2^ = 0%
		Herbal tea	1	25	–0.93 [–8.93, 7.07]	*P* = 0.82	Not applicable
	Intervention duration	<3 months	3	130	–2.75 [–5.50, 0.01]	*P* = 0.05	I^2^ = 0%
		≥3 months	2	64	–2.62 [–6.48, 1.24]	*P* = 0.18	I^2^ = 0%
	Race of participants	Asian	1	34	–3.40 [–9.27, 2.47]	*P* = 0.26	Not applicable
		Caucasian	4	160	–2.59 [–5.01, –0.16]	*P* = 0.04	I^2^ = 0%
BMI	Race of participants	Asian	1	34	–0.20 [–1.64, 1.24]	*P* = 0.79	Not applicable
		Caucasian	2	75	–0.47 [–2.36, 1.42]	*P* = 0.62	I^2^ = 0%
WHR	Race of participants	Asian	1	34	–0.04 [–0.07, –0.01]	*P* = 0.004	Not applicable
		Caucasian	2	75	–0.00 [–0.05, 0.04]	*P* = 0.89	I^2^ = 31%
BFR	Race of participants	Asian	1	34	0.60 [–3.09, 4.29]	*P* = 0.75	Not applicable
		Caucasian	1	45	–0.29 [–4.41, 3.83]	*P* = 0.89	Not applicable
FBG	Tea type	Green tea	1	34	–0.40 [–0.62, –0.18]	*P* = 0.0003	Not applicable
		Herbal tea	1	25	–0.37 [–0.86, 0.12]	*P* = 0.14	Not applicable
	Intervention duration	<3 months	1	25	–0.37 [–0.86, 0.12]	*P* = 0.14	Not applicable
		≥3 months	1	34	–0.40 [–0.62, –0.18]	*P* = 0.0003	Not applicable
	Race of participants	Asian	1	34	–0.40 [–0.62, –0.18]	*P* = 0.0003	Not applicable
		Caucasian	1	25	–0.37 [–0.86, 0.12]	*P* = 0.14	Not applicable
FINS	Tea type	Green tea	2	94	–3.71 [–5.25, –2.17]	*P <*0.00001	I^2^ = 0%
		Herbal tea	1	25	–2.19 [–5.19, 0.81]	*P* = 0.15	Not applicable
	Intervention duration	<3 months	2	85	–3.42 [–4.80, –2.04]	*P <*0.00001	I^2^ = 0%
		≥3 months	1	34	–1.70 [–13.20, 9.80]	*P* = 0.77	Not applicable
	Race of participants	Asian	1	34	–1.70 [–13.20, 9.80]	*P* = 0.77	Not applicable
		Caucasian	2	85	–3.42 [–4.80, –2.04]	*P* < 0.00001	I^2^ = 0%
FT	Tea type	Green tea	1	60	–0.72 [–1.09, –0.35]	*P* = 0.0001	Not applicable
		Herbal tea	1	41	–0.18 [–0.34, –0.02]	*P* = 0.03	Not applicable
TT	Tea type	Green tea	1	34	0.04 [–0.29, 0.37]	*P* = 0.81	Not applicable
		Herbal tea	2	66	–0.11 [–0.32, 0.10]	*P* = 0.30	I^2^ = 34%
	Race of participants	Asian	1	34	0.04 [–0.29, 0.37]	*P* = 0.81	Not applicable
		Caucasian	2	66	–0.11 [–0.32, 0.10]	*P* = 0.30	I^2^ = 34%
DHEAS	Tea type	Green tea	1	34	–0.14 [–0.78, 0.50]	*P* = 0.67	Not applicable
		Herbal tea	2	66	–0.09 [–0.70, 0.52]	*P* = 0.78	I^2^ = 0%
	Race of participants	Asian	1	34	–0.14 [–0.78, 0.50]	*P* = 0.67	Not applicable
		Caucasian	2	66	–0.09 [–0.70, 0.52]	*P* = 0.78	I^2^ = 0%
LH	Tea type	Green tea	1	34	–0.30 [–2.61, 2.01]	*P* = 0.80	Not applicable
		Herbal tea	2	66	–0.53 [–6.97, 5.92]	*P* = 0.87	I^2^ = 69%
	Race of participants	Asian	1	34	–0.30 [–2.61, 2.01]	*P* = 0.80	Not applicable
		Caucasian	2	66	–0.53 [–6.97, 5.92]	*P* = 0.87	I^2^ = 69%
FSH	Tea type	Green tea	1	34	1.00 [0.28, 1.72]	*P* = 0.007	Not applicable
		Herbal tea	2	66	0.01 [–1.04, 1.06]	*P* = 0.98	I^2^ = 6%
	Intervention duration	<3 months	2	66	0.01 [–1.04, 1.06]	*P* = 0.98	I^2^ = 6%
		≥3 months	1	34	1.00 [0.28, 1.72]	*P* = 0.007	Not applicable
	Race of participants	Asian	1	34	1.00 [0.28, 1.72]	*P* = 0.007	Not applicable
		Caucasian	2	66	0.01 [–1.04, 1.06]	*P* = 0.98	I^2^ = 6%

Meta-analysis of three studies (23, 27, 28) that used green tea as the intervention were included to assess the effect of tea supplements on BMI and WHR. There was no significant difference in BMI (WMD −0.30, 95% CI −1.45 to 0.85, *P* = 0.61, I^2^ = 0%, **Figure 3B**) and WHR (WMD −0.02, 95% CI −0.06 to 0.02, *P* = 0.30, I^2^ = 62%, **Figure 3C**) between the groups. The subgroup analysis showed that tea was statistically significant in reducing WHR in Asian PCOS patients (WMD −0.04, 95% CI −0.07 to −0.01, *P* = 0.004, I^2^ =/, **Table 3**).

BFR was evaluated in two studies (23, 27) totaling 79 patients that compared tea supplements with placebo. There was not strong evidence that the tea supplements had an effect on decreasing BFR because of no statistical difference (WMD 0.20, 95% CI −2.54 to 2.95, *P* = 0.88, I^2^ = 0%, **Figure 3D**).

WC and HC were measured in two studies (27, 28) that compared tea supplements with placebo, and these two studies were included in the meta-analysis. We did not observe a significant decrease in WC (WMD −0.64, 95% CI −5.22 to 3.94, *P* = 0.79, I^2^ = 0%, **Figure 3E**) and HC (WMD −1.53, 95% CI −6.87 to 3.82, *P* = 0.58, I^2^ = 31%, **Figure 3F**) between tea and placebo.

### Effect of Tea Supplements on Metabolic Indexes

**Figure 4:**
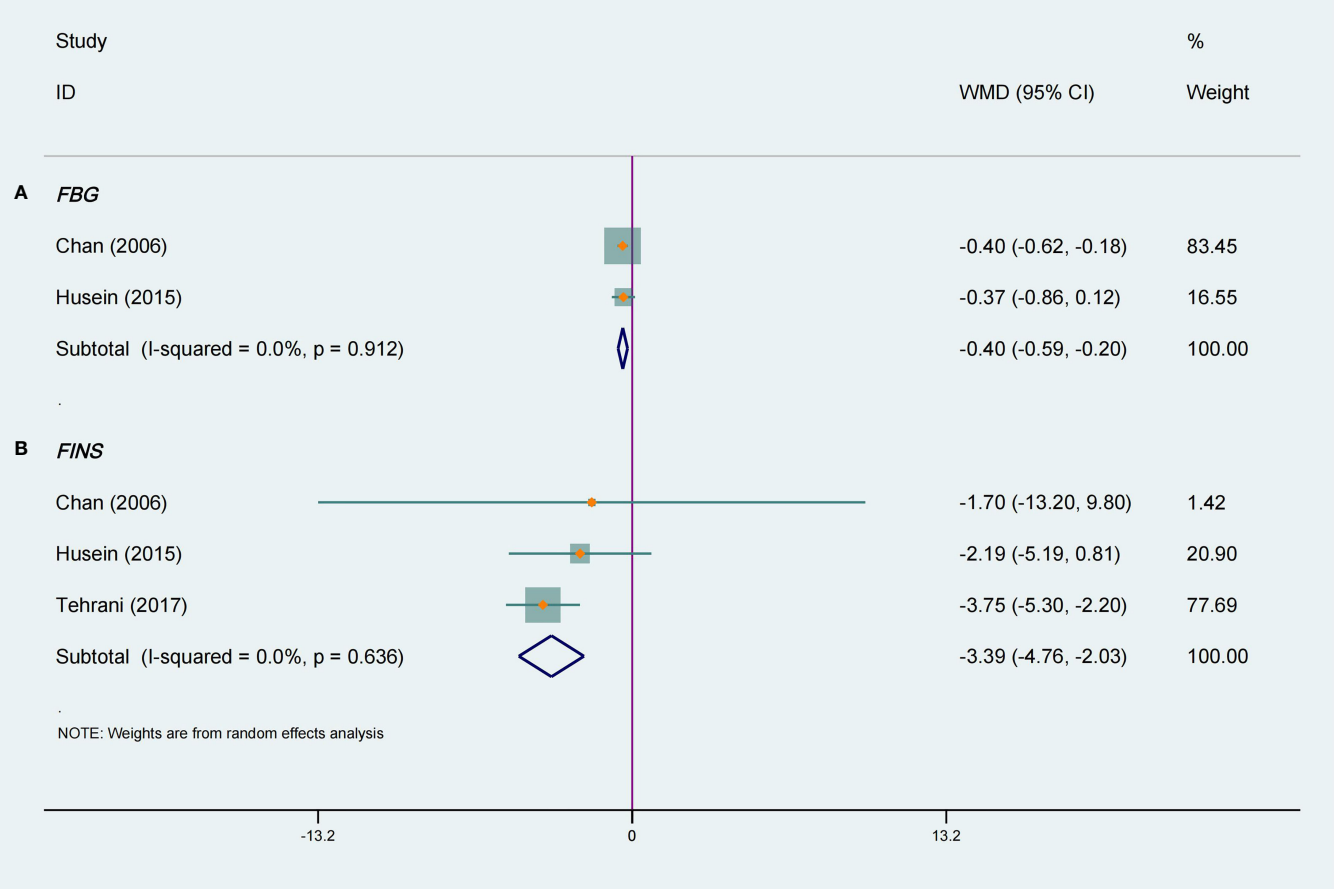
Meta-analyses of the effect of tea supplements on metabolic parameters. **(A)** FBG and **(B)** FINS.

**Figure 4A** shows the meta-analysis of FBG in two RCTs (18, 23) with a total of 59 participants. The pooled results were statistically significant, and compared with the placebo group tea supplements had a positive effect on FBG in women with PCOS (WMD −0.40, 95% CI −0.59 to −0.20, *P* < 0.0001, I^2^ = 0%). In the stratified analysis, interventions lasting 3 months or more had a significantly great effect on FBG levels (WMD −0.40, 95% CI −0.62 to −0.18, *P* = 0.0003, I^2^ =/, **Table 3**). Green tea had a considerable effect on FBG levels (WMD −0.40, 95% CI −0.62 to −0.18, *P* = 0.0003, I^2^ =/), but herbal tea did not (**Table 3**). Moreover, it can be observed that tea was effective in lowering FBG in Asian PCOS female (WMD −0.40, 95% CI −0.62 to −0.18, *P* = 0.0003, I^2^ =/, **Table 3**), but not in Caucasians.

Three studies (14, 18, 23) totaling 119 patients were included in a meta-analysis that evaluated the effects on FINS. In terms of ameliorating FINS, there was a significant difference between the tea supplements group and the placebo group, and there was no heterogeneity among the included studies (WMD −3.40, 95% CI −4.76 to −2.03, *P* < 0.00001, I^2^ = 0%, **Figure 4B**). The stratified analysis suggested that green tea can reduce FINS levels (WMD −3.71, 95% CI −5.25 to −2.17, *P* < 0.00001, I^2^ = 0%, **Table 3**), but herbal tea cannot. Furthermore, tea supplementation had a significant effect on FINS levels when intervention lasting less than 3 months (WMD −3.42, 95% CI −4.80 to −2.04, *P* < 0.0001, I^2^ = 0%, **Table 3**). The results of the subgroup analysis revealed a significant reduction in FINS in Caucasian participants (WMD −3.42, 95% CI −4.80 to −2.04, *P* < 0.00001, I^2^ = 0%, **Table 3**), but not in Asians.

### Effect of Tea Supplements on Hormone Parameters

**Figure 5:**
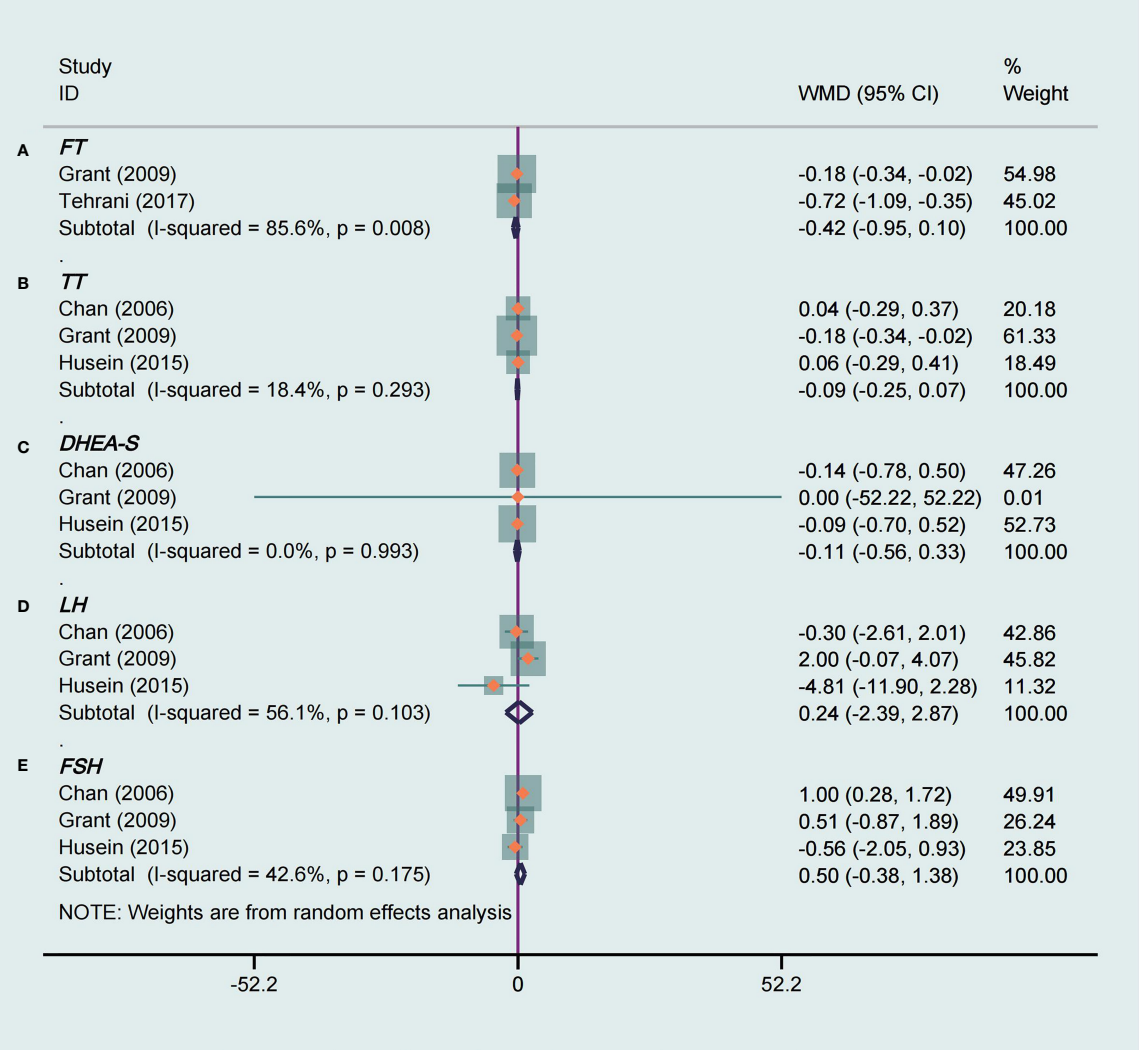
Meta-analyses of the effect of tea supplements on hormone parameters. **(A)** FT, **(B)** TT, **(C)** DHEA-S, **(D)** LH, and **(E)** FSH.

Only two RCTs (14, 17) reported FT as an outcome measure. There was no significant difference in FT of the intervention groups when compared with the placebo groups, and with high heterogeneity (WMD −0.42, 95% CI −0.95 to 0.10, *P* = 0.12, I^2^ = 86%, **Figure 5A**). However, the results of subgroup analysis suggested green tea (WMD -0.72, 95% CI -1.09 to -0.35, *P* = 0.0001, I^2^ =/, **Table 3**) and herbal tea (WMD -0.18, 95% CI -0.34 to -0.02, *P* = 0.003, I^2^ =/, **Table 3**) both had significant effect on decreasing FT.

Three studies (17, 18, 23) totaling 100 patients were included that evaluated the influence of tea supplements on TT, DHEAS, LH, and FSH. For TT and DHEAS, and we found no significant decrease on TT (WMD −0.09, 95% CI −0.25 to 0.07, *P* = 0.26, I^2^ = 18%, **Figure 5B**) and DHEAS (WMD −0.11, 95% CI -0.56 to 0.33, *P* = 0.62, I^2^ = 0%, **Figure 5C**) levels compared with placebo groups. There was no statistically significant effect of tea on LH (WMD 0.24, 95% CI –2.39 to 2.87, *P* = 0.86, I^2^ = 56%, **Figure 5D**). In addition, tea had no significant effect on improving FSH level in PCOS patients, and with mild heterogeneity (WMD 0.50, 95% CI -0.38 to 1.38, *P* = 0.27, I^2^ = 43%, **Figure 5E**). The subgroup analysis indicated that interventions with tea supplements for 3 months or longer had a beneficial effect on FSH (WMD 1.00, 95% CI 0.28 to 1.72, *P* = 0.007, I^2^ =/, **Table 3**). There was no significant effect for the intervention with herbal tea, but there was a significant effect with green tea (WMD 1.00, 95% CI 0.28 to 1.72, *P* = 0.007, I^2^ =/, **Table 3**). In the subgroup analysis based on ethnicity, the results showed that the FSH levels were significantly decreased in Asian PCOS patients (WMD 1.00, 95% CI 0.28 to 1.72, *P* = 0.007, I^2^ =/, **Table 3**) after tea supplements intervention but not in Caucasian participants.

### Adverse Events

**Figure 6:**
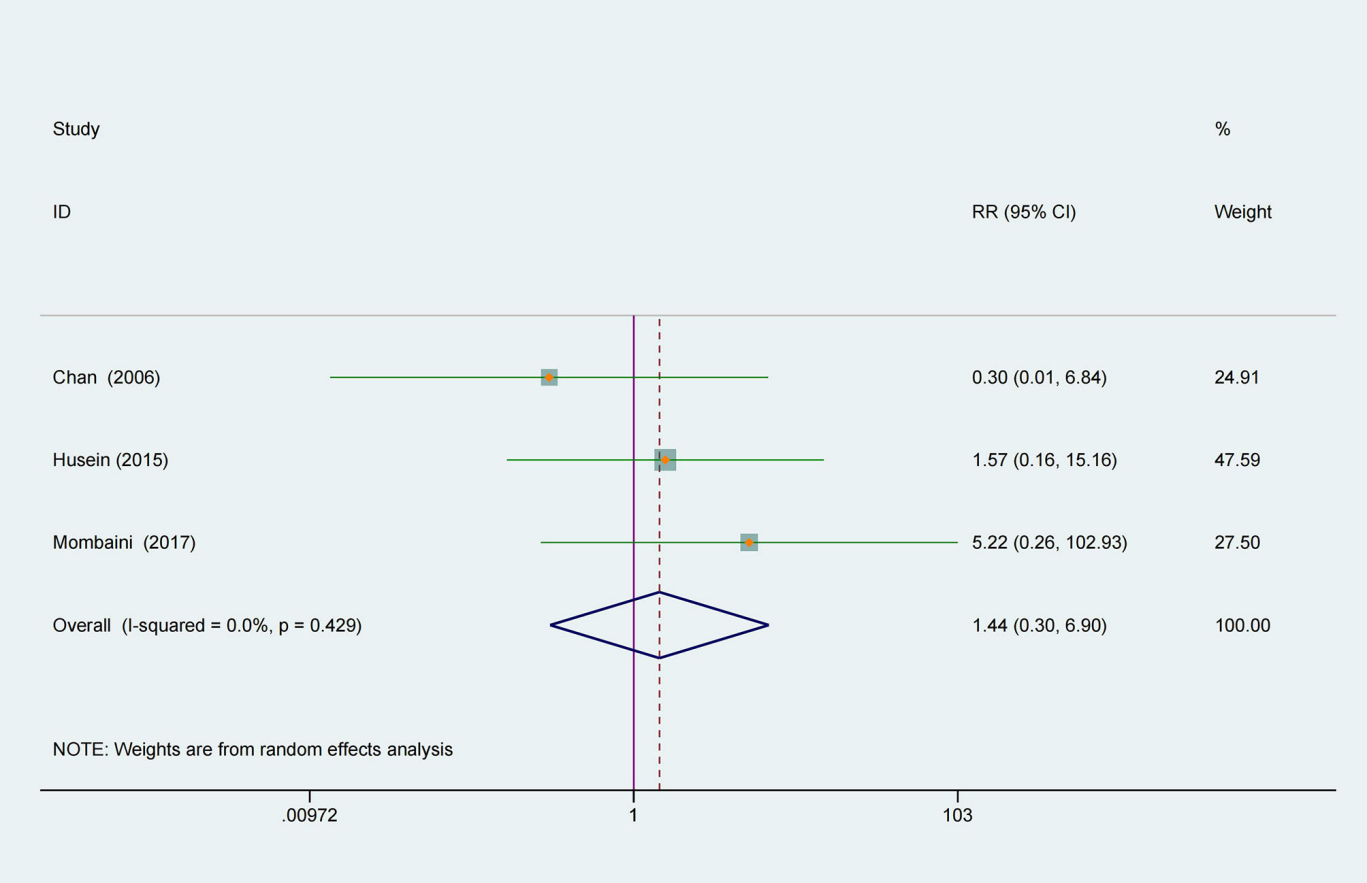
Effect of tea supplements on the risk of adverse events.

The meta-analysis of adverse events showed that tea supplement was not more likely to cause adverse events, it may be a safe treatment (RR 1.45, 95% CI 0.30 to 6.90, *P* = 0.65, I^2^ = 0%, **Figure 6**).

### Publication Bias

**Figure 7:**
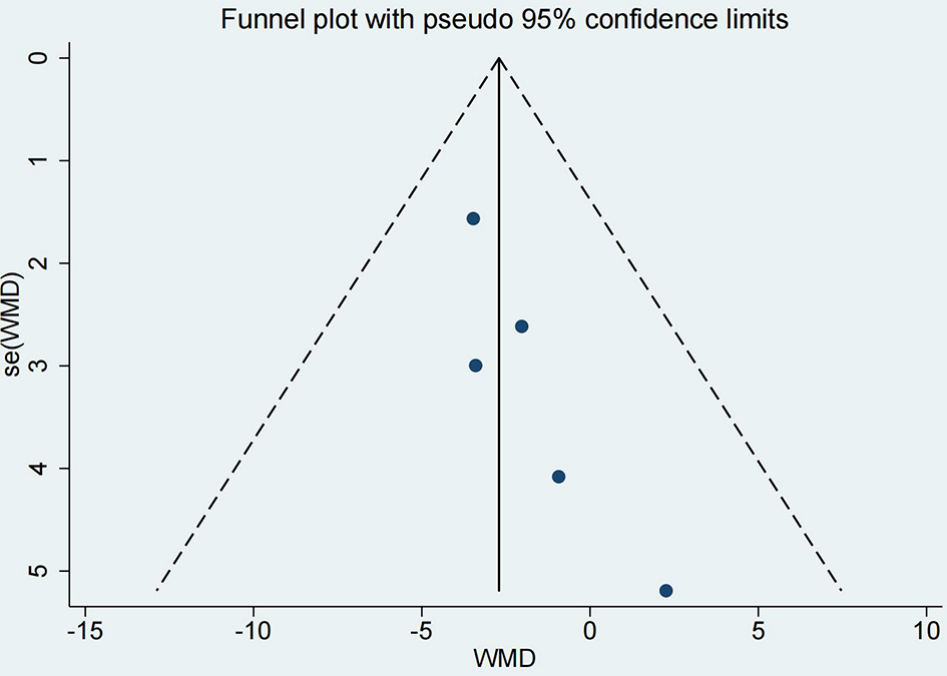
The funnel plot of the literatures.

In order to better evaluate the results of our study, although there were less than 10 articles included in this study, the funnel plot was used to evaluate publication bias. Visual assessment of the funnel plot and Egger’s test all indicated that there was no significant publication bias (*P* = 0.058, **Figure 7**).

### Sensitivity Analysis

Significant heterogeneity was observed among the studies reporting WHR, FT and LH, so sensitivity analysis was conducted to explore the potential causes. The results of sensitivity analysis indicated that the pooled WMD was not substantially altered when any single study was excluded, suggesting that the results of the current meta-analysis are robust (***Supplementary Appendix 3***, ***Appendix 4***, ***Appendix 5***).

## Discussion

### Main Findings

The meta-analysis of the six included studies revealed improvements in several primary and secondary outcome measures with low heterogeneity. Compared with the placebo, tea supplements reduced weight and improved IR (reduced FBG and FINS) in women with PCOS.

The six studies included in this meta-analysis used green tea, marjoram tea, and spearmint herbal tea as intervention methods, the intervention durations and races of participants were not exactly the same. The subgroup analyses suggested that different kinds of tea and intervention duration had different effects on body weight, FBG, FINS, and FSH in PCOS patients. It is clear from the included studies that green tea rich in tea polyphenols can significantly improve the above clinical outcomes. And green tea and herbal tea are both effective in reducing FT levels. As for the duration of intervention, long-term intervention (up to 3 months or more) can have a significant effect on FBG and FSH. An interesting finding was that improving FINS was statistically significant in trials lasting less than 3 months but were not significant in trials that lasted 3 months. In the subgroup analysis performed according to ethnicity, we observed that tea had no effect on reducing WHR, FSH and FBG in Caucasians PCOS patients, but it was effective in Asians. And reducing body weight and FINS in Caucasians participants was statistically significant, but not in Asians.

### Meaning of the Study for Possible Clinical Use

Overweight or obesity is seen in 30%–60% of patients with PCOS (29), meanwhile, obesity is also one of the possible factors leading to PCOS (3). Overweight women with PCOS have more serious endocrine hormone disorders and metabolic dysfunction, which can lead to hyperandrogenemia and IR (30, 31). One study suggested that the prevalence of PCOS in overweight and obese women was 28.3%, which was clearly higher than the prevalence in women with normal body weight (5.5%) (32). Obesity may also affect endometrial receptivity (33), and the fertility of obese PCOS patients was lower than that of normal-weight PCOS patients. An epidemiological study of PCOS women suggested that the incidence of infertility is higher in obese patients than in non-obese patients (34). Common treatments for obesity in PCOS patients include pharmaceutical treatments and lifestyle interventions, such as diet intervention and physical activity. However, many western medicines have significant side effects. For example, studies have shown that orlistat may lead to a series of gastrointestinal adverse reactions and cause fat-soluble vitamin deficiencies (35, 36). Based on this meta-analysis, tea supplements as a dietary intervention may be recommended for the treatment of PCOS patients with overweight and IR. Green tea catechins can inhibit adipocyte differentiation and proliferation and can reduce various digestive enzymes to prevent excessive carbohydrate and fat absorption, thus having anti-obesity effects (20). An animal trial showed that there was a significant reduction in the body weight and triglyceride levels after 8 weeks of intervention in the catechins group but not in the control group (37). One intervention trial showed that green tea exact may play a role in enhancing body energy consumption by sympathetic activation of thermogenesis and fat oxidation (38). Taken together, tea supplements have been shown to be effective in reducing weight in patients with PCOS. Our study did not find significant effects of tea on BMI, BFR, WHR, WC, or HC in patients with PCOS. However, Mombaini et al.’s (27) study suggested that BMI and BFR were decreased in PCOS patients after taking green tea tablets for 45 days. Additionally, Farhadian et al. (28) also found that the trend for reduction of BMI, WC, and HC in the green tea group was statistically significant. We cannot deny the positive effect of tea supplements on the body parameters of PCOS patients based on the negative results of this analysis, which may be due to the lack of appropriate intervention time, and thus it is still possible that tea supplements may be a safe and inexpensive treatment for losing weight.

Abnormal glucose metabolism and IR are both driving factors of type 2 diabetes mellitus in women with PCOS (39), and there is evidence that secondary hyperinsulinemia may aggravate metabolic disorders, hyperandrogenism, or irregular menstruation in patients with PCOS (35). Compared with the control group, it was observed that tea supplements significantly decreased FBG and FINS levels in PCOS patients in this meta-analysis, which suggested that tea or tea extracts may have an effect on improving glucose metabolism function. The bioactive components in tea, including polysaccharides and polyphenols, play a role in inhibiting the increase in blood glucose seen in PCOS patients with IR. Tea polyphenols have a hypoglycemic effect mainly by reducing the intake of exogenous sugar, promoting the utilization and transformation of sugar in the body, protecting islet β cells, and promoting insulin receptor substrate function (40). An experimental animal study found that blood triglycerides, cholesterol, low density lipoprotein, FBG, FINS, and the levels of free fatty acids in the tea polyphenols intervention group were lower than those in the control group (41). In a previous meta-analysis on the effects of green tea on insulin sensitivity and glycemic control, there was no decrease in FBG and FINS. This result was contrary to the findings of many animal experiments, and the authors suggested that this might be because of the low dose of EGCG, the short treatment time, variations in green tea composition, and species-specific differences in glucose metabolism (21). Consistent with our research, in a short-term crossover trial it was observed that the plasma glucose of participants who drank black tea was lowered at 120 min compared with the control group and the caffeinated group. It presented an idea about the hypoglycemic mechanism of black tea, which might be by inhibiting intestinal glucose transport and enhancing insulin secretion (42). The results of our meta-analysis support the view that tea may be a preventive strategy to reduce the risk of diabetes mellitus in patients with PCOS, but further studies are needed to confirm the evidence.

Hyperandrogenemia is the main pathological feature of PCOS, with menstrual irregularity, acne, hirsutism, and other clinical signs, and it is closely related to the development of IR and metabolic syndrome. The pathogenesis may include abnormal gonadotropin secretion and hyperinsulinism caused by IR. Excessive LH can promote the proliferation of ovarian stroma and thecal cells and can also lead to excessive androgen secretion, including testosterone and androstenedione (43). High insulin levels promote the P450C17α enzyme system, which is used to synthesize androgens in the ovaries and to up-regulate the LH receptor in the follicular membrane cells in the ovary, thus enhancing the androgen-promoting effect of LH (44). In the present meta-analysis, although there was insufficient evidence that tea had beneficial effect on female reproductive hormones, some findings provided evidence for further exploration. The within-group data in one RCT suggested that FT and TT levels and subjective assessments of hirsutism scored by the DLQI were significantly reduced in the spearmint tea group after the intervention (17). It can thus be seen that tea has potential effects on lowering androgen levels in patients with PCOS. Most of the analysis results of our research are negative, but the regulatory effect of tea on various reproductive hormones in PCOS patients cannot be ruled out.

Four studies (17, 18, 23, 27) reported on adverse reactions, one of which reported no adverse reactions (17), while the other two studies did not mention anything about adverse events (14, 28). In one trial, there was no gastrointestinal discomfort in the intervention group, but it was present in the placebo group, and this may have been caused by the capsule shell (23). One study (18) reported more frequent urination in both the placebo group and the intervention group, which may be because the intervention measure was to drink two cups of tea (250ml each) every day. Two studies both reported adverse gastrointestinal reactions, but the number of events was small and symptoms were mild (18, 27). According to the current evidence, tea supplements appear to be a relatively safe treatment on the whole, but more long-term and high-quality RCTs are needed in order to further evaluate the safety of tea supplements.

### Strengths and Limitations

To the best of our knowledge, this study is the first comprehensive meta-analysis of all available RCTs assessing the effectiveness and safety of tea supplements for patients with PCOS. And we conducted subgroup analyses to evaluate the clinical efficacy of different types of tea and different duration of intervention, and the efficacy of tea supplements in individuals of differing race.

While the curative effect of tea supplements was seen in this meta-analysis, there are some limitations that must be considered. First, the duration of the studies included in the meta-analysis was generally short, ranging from 1 to 3 months. Tea supplements may require longer treatment duration if they are to have an effect on anthropometric parameters (BFR, WC and HC) and reproductive hormone levels. Second, we did not consider the differences in tea supplement intake, the dosage form, or the dosage of tea supplements, which were not uniform in the included trials and may have caused variations in the clinical outcomes. Third, although the included trials were mostly of high quality, the amounts of articles and participants was not large enough. In addition, the included participants were mostly characterized by obesity, and the treatment effects on all types of PCOS need further research. In addition, the types of tea as intervention were relatively single.

We observed significant heterogeneity among the studies reporting WHR, FT and LH. And we sequentially excluded each study to perform subgroup analysis. Heterogeneity of WHR decreased from 62% to 0% after eliminating the study by Farhadian et al., it may be due to differences in random allocation methods, the block randomization method in this study was based on age and BMI. When the study by Grant was excluded, heterogeneity of LH decreased from 56% to 29%, it indicated that type of tea may be factor affecting heterogeneity. The high heterogeneity of FT may be due to the types, dosage forms and doses of tea and duration time in the two studies were all different.

### Implications for Future

This study found that tea supplements may reduce weight, FBG and FINS in patients with PCOS. In the aspect of improving hormone parameters may not have an advantage over the control group, but we cannot absolutely deny the efficacy of tea in regulating reproductive hormone levels due to the relatively small sample size. Therefore, more large-scale RCTs are needed in order to evaluate the real effect of tea on PCOS. In addition, tea supplements have unique value in the complementary treatment of PCOS. Various types of tea can cater to the different taste preferences of PCOS patients and provide more choices for them. Some studies have shown that several tea extracts also have positive effects on weight loss and glucose and lipid metabolism, such as oolong, black, and Pu’er tea (45, 46). Thus, future studies on tea for the treatment of PCOS could use different types of tea as interventions in order to find more kinds of tea supplements with therapeutic effect on PCOS.

## Conclusion

This systematic review and meta-analysis provides evidence that tea supplements as a dietary intervention may be a possible treatment for patients with PCOS From what had been discussed above, tea supplements may have a positive therapeutic effect on PCOS patients with obesity and IR, but the regulatory effect on hyperandrogenemia remains to be further studied. We expect more clinical trials to explore the clinical efficacy of tea on PCOS.

## Data Availability Statement

The original contributions presented in the study are included in the article/**Supplementary Material**. Further inquiries can be directed to the corresponding authors.

## Author Contributions

WS and YZ conceptualized the research question. YP and ZZ participated in drafting and writing the review. YP, BJ and ZZ participated in the formulation of retrieval strategies, data acquisition, data analysis and quality assessment. TY and YQ participated in the drawing of tables and figures. MH and XY participated in critical revision of the manuscript. All authors contributed to the research and approved the final manuscript.

## Funding

This work is supported by the Young Scientists Project of the National Natural Science Foundation of China (81803945), Scientific Research Project of Traditional Chinese Medicine in Heilongjiang Province (ZHY19024), and the Project of Young Innovative Talents in Colleges and Universities in Heilongjiang Province (UNPYSCT-2016216).

## Conflict of Interest

The authors declare that the research was conducted in the absence of any commercial or financial relationships that could be construed as a potential conflict of interest.

## Publisher’s Note

All claims expressed in this article are solely those of the authors and do not necessarily represent those of their affiliated organizations, or those of the publisher, the editors and the reviewers. Any product that may be evaluated in this article, or claim that may be made by its manufacturer, is not guaranteed or endorsed by the publisher.

## Abbreviations

PCOS, polycystic ovary syndrome; RCTs, randomized controlled trials; WMDs, Weighted mean differences; RRs, rick ratios; CI, confidence interval; BFR, body fat rate; FINS, fasting insulin; IR, insulin resistance; FT, free testosterone; TT, total testosterone; BMI, body mass index; TG, triglycerides; FSH, follicle-stimulating hormone; HC, hip circumference; SHBG, sex hormone binding globulin; LH, luteinizing hormone; WC, waist circumference; WHR, waist-to-hip ratio; HbA1c, glycated hemoglobin; FBG, fasting blood glucose; FG, Ferriman-Gallwey score; EGCG, epigallocatechin gallate; DLQI, dermatology quality of life index; hsCRP, high-sensitivity C-reactive protein; FAI, free androgen index; A2, androstenedione; P, progesterone; E2, estradiol; FLP, fasting leptin; IL-6, interleukin 6; HDL, high-density lipoprotein; TNF-α, tumor necrosis factor-α; LDL, low-density lipoprotein; non-HDL-C, fasting non-HDL cholesterol; DHEAS, dehydroepiandrosterone sulfate; GIR, fasting blood glucose/fasting insulin ratio; HOMA-IR, homeostasis model assessment of insulin resistance; CBM, Chinese Biomedical Literature Database; CNKI, Chinese National knowledge Infrastructure; PICOS, participants; intervention; control; outcome and study design; PRISMA, Preferred Reporting Items for Systematic Reviews and Meta-Analyses.
