# Supplementary material for: Effects of Tea Consumption on Anthropometric Parameters, Metabolic Indexes and Hormone Levels of Women with Polycystic Ovarian Syndrome: A Systematic Review and Meta-Analysis of Randomized Controlled Trials
Source: Front Endocrinol (Lausanne). 2021 Dec 13;12:736867. doi: 10.3389/fendo.2021.736867 (PMC8710535; doi:10.3389/fendo.2021.736867)
Supplement: Supplementary file 1 [file DataSheet_1.pdf]

**Supplementary Appendix 1 Details of Search Strategy Source: PubMed; Searched on: September 2021; Results: 19**

| Search | Query |
|---|---|
| #1 | "Polycystic Ovary Syndrome"[Mesh] |
| #2 | Polycystic Ovary Syndrome[Title/Abstract] |
| #3 | Ovary Syndrome, Polycystic[Title/Abstract] |
| #4 | Syndrome, Polycystic Ovary[Title/Abstract] |
| #5 | Stein-Leventhal Syndrome[Title/Abstract] |
| #6 | Stein Leventhal Syndrome[Title/Abstract] |
| #7 | Syndrome, Stein-Leventhal[Title/Abstract] |
| #8 | Sclerocystic Ovarian Degeneration[Title/Abstract] |
| #9 | Ovarian Degeneration, Sclerocystic[Title/Abstract] |
| #10 | Sclerocystic Ovary Syndrome[Title/Abstract] |
| #11 | Polycystic Ovarian Syndrome[Title/Abstract] |
| #12 | Ovarian Syndrome, Polycystic[Title/Abstract] |
| #13 | Polycystic Ovary Syndrome 1[Title/Abstract] |
| #14 | Sclerocystic Ovaries[Title/Abstract] |
| #15 | Ovary, Sclerocystic[Title/Abstract] |
| #16 | Sclerocystic Ovary[Title/Abstract] |
| #17 | PCOS[Title/Abstract] |
| #18 | #1 OR #2 OR #3 OR #4 OR #5 OR #6 OR #7 OR #8 OR #9 OR #10 OR #11 OR #12 OR #13 OR #14 OR #15 OR #16 OR #17 |
| #19 | "Tea"[Mesh] |
| #20 | Tea[Title/Abstract] |
| #21 | Black Tea[Title/Abstract] |
| #22 | Black Teas[Title/Abstract] |
| #23 | Tea, Black[Title/Abstract] |
| #24 | Teas, Black[Title/Abstract] |
| #25 | Green Tea[Title/Abstract] |
| #26 | Green Teas[Title/Abstract] |
| #27 | Tea, Green[Title/Abstract] |
| #28 | Teas, Green[Title/Abstract] |
| #29 | "Teas, Herbal"[Mesh] |
| #30 | Teas, Herbal[Title/Abstract] |
| #31 | Herbal Tea[Title/Abstract] |
| #32 | Tea, Herbal[Title/Abstract] |
| #33 | Tisanes[Title/Abstract] |
| #34 | Tisane[Title/Abstract] |
| #35 | Herbal Teas[Title/Abstract] |
| #36 | #19 OR #20 OR #21 OR #22 OR #23 OR #24 OR #25 OR #26 OR #27 OR #28 OR #29 OR #30 OR #31 OR #32 OR #33 OR #34 OR #35 |
| #37 | #18 AND #36 |

**Source: Cochrane Library; Searched on: September 2021; Results: 19**

| Search | Query |
|---|---|
| #1 | MeSH descriptor: [Polycystic Ovary Syndrome] explode all trees |
| #2 | (Polycystic Ovary Syndrome):ti,ab,kw |
| #3 | (Ovary Syndrome, Polycystic):ti,ab,kw |
| #4 | (Syndrome, Polycystic Ovary):ti,ab,kw |
| #5 | (Stein-Leventhal Syndrome):ti,ab,kw |
| #6 | (Stein Leventhal Syndrome):ti,ab,kw |
| #7 | (Syndrome, Stein-Leventhal):ti,ab,kw |
| #8 | (Sclerocystic Ovarian Degeneration):ti,ab,kw |
| #9 | (Ovarian Degeneration, Sclerocystic):ti,ab,kw |
| #10 | (Sclerocystic Ovary Syndrome):ti,ab,kw |
| #11 | (Polycystic Ovarian Syndrome):ti,ab,kw |
| #12 | (Ovarian Syndrome, Polycystic):ti,ab,kw |
| #13 | (Polycystic Ovary Syndrome 1):ti,ab,kw |
| #14 | (Sclerocystic Ovaries):ti,ab,kw |
| #15 | (Ovary, Sclerocystic):ti,ab,kw |
| #16 | (Sclerocystic Ovary):ti,ab,kw |
| #17 | (PCOS):ti,ab,kw |
| #18 | #1 OR #2 OR #3 OR #4 OR #5 OR #6 OR #7 OR #8 OR #9 OR #10 OR #11 OR #12 OR #13 OR #14 OR #15 OR #16 OR #17 |
| #19 | MeSH descriptor: [Tea] explode all trees |
| #20 | (Tea):ti,ab,kw |
| #21 | (Black Tea):ti,ab,kw |
| #22 | (Black Teas):ti,ab,kw |
| #23 | (Tea, Black):ti,ab,kw |
| #24 | (Teas, Black):ti,ab,kw |
| #25 | (Green Tea):ti,ab,kw |
| #26 | (Green Teas):ti,ab,kw |
| #27 | (Tea, Green):ti,ab,kw |
| #28 | (Teas, Green):ti,ab,kw |
| #29 | MeSH descriptor: [Teas, Herbal] explode all trees |
| #30 | (Teas, Herbal):ti,ab,kw |
| #31 | (Herbal Tea):ti,ab,kw |
| #32 | (Tea, Herbal):ti,ab,kw |
| #33 | (Tisanes):ti,ab,kw |
| #34 | (Tisane):ti,ab,kw |
| #35 | (Herbal Teas):ti,ab,kw |
| #36 | #19 OR #20 OR #21 OR #22 OR #23 OR #24 OR #25 OR #26 OR #27 OR #28 OR #29 OR #30 OR #31 OR #32 OR #33 OR #34 OR #35 |
| #37 | #18 AND #36 |

**Source: Embase; Searched on: September 2021; Results: 42**

| Search | Query |
|---|---|
| #1 | 'ovary polycystic disease'/exp |
| #2 | 'polycystic ovary syndrome':ab,ti |
| #3 | 'ovary syndrome, polycystic':ab,ti |
| #4 | 'syndrome, polycystic ovary':ab,ti |
| #5 | 'stein-leventhal syndrome':ab,ti |
| #6 | 'stein leventhal syndrome':ab,ti |
| #7 | 'syndrome, stein-leventhal':ab,ti |
| #8 | 'sclerocystic ovarian degeneration':ab,ti |
| #9 | 'ovarian degeneration, sclerocystic':ab,ti |
| #10 | 'sclerocystic ovary syndrome':ab,ti |
| #11 | 'polycystic ovarian syndrome':ab,ti |
| #12 | 'ovarian syndrome, polycystic':ab,ti |
| #13 | 'polycystic ovary syndrome 1':ab,ti |
| #14 | 'sclerocystic ovaries':ab,ti |
| #15 | 'ovary, sclerocystic':ab,ti |
| #16 | 'sclerocystic ovary':ab,ti |
| #17 | 'pcos':ab,ti |
| #18 | #1 OR #2 OR #3 OR #4 OR #5 OR #6 OR #7 OR #8 OR #9 OR #10 OR #11 OR #12 OR #13 OR #14 OR #15 OR #16 OR #17 |
| #19 | 'tea'/exp |
| #20 | tea:ab,ti |
| #21 | 'black tea':ab,ti |
| #22 | 'black teas':ab,ti |
| #23 | 'tea, black':ab,ti |
| #24 | 'teas, black':ab,ti |
| #25 | 'green tea':ab,ti |
| #26 | 'green teas':ab,ti |
| #27 | 'tea, green':ab,ti |
| #28 | 'teas, green':ab,ti |
| #29 | 'Teas, Herbal'/exp |
| #30 | 'Teas, Herbal':ab,ti |
| #31 | 'Herbal Tea':ab,ti |
| #32 | 'Tea, Herbal':ab,ti |
| #33 | 'Tisanes':ab,ti |
| #34 | 'Tisane':ab,ti |
| #35 | 'Herbal Teas':ab,ti |
| #36 | #19 OR #20 OR #21 OR #22 OR #23 OR #24 OR #25 OR #26 OR #27 OR #28 OR #29 OR #30 OR #31 OR #32 OR #33 OR #34 OR #35 |
| #37 | #18 AND #36 |

**Source: Web of Science; Searched on: September 2021; Results: 29**

| Search | Query |
|---|---|
| #1 | TS="polycystic ovary syndrome" |
| #2 | TS="Ovary Syndrome, Polycystic" |
| #3 | TS="Syndrome, Polycystic Ovary" |
| #4 | TS="Stein-Leventhal Syndrome" |
| #5 | TS="Stein Leventhal Syndrome" |
| #6 | TS="Syndrome, Stein-Leventhal" |
| #7 | TS="Sclerocystic Ovarian Degeneration" |
| #8 | TS="Ovarian Degeneration, Sclerocystic" |
| #9 | TS="Sclerocystic Ovary Syndrome" |
| #10 | TS="Polycystic Ovarian Syndrome" |
| #11 | TS="Ovarian Syndrome, Polycystic" |
| #12 | TS="Polycystic Ovary Syndrome 1" |
| #13 | TS="Sclerocystic Ovaries" |
| #14 | TS="Ovary, Sclerocystic" |
| #15 | TS="Sclerocystic Ovary" |
| #16 | TS=PCOS |
| #17 | #1 OR #2 OR #3 OR #4 OR #5 OR #6 OR #7 OR #8 OR #9 OR #10 OR #11 OR #12 OR #13 OR #14 OR #15 OR #16 |
| #18 | TS="tea" |
| #19 | TS="Black Tea" |
| #20 | TS="Black Teas" |
| #21 | TS="Tea, Black" |
| #22 | TS="Teas, Black" |
| #23 | TS="Green Tea" |
| #24 | TS="Green Teas" |
| #25 | TS="Tea, Green" |
| #26 | TS="Teas, Green" |
| #27 | TS="Teas, Herbal" |
| #28 | TS="Herbal Tea" |
| #29 | TS="Tea, Herbal" |
| #30 | TS="Tisanes" |
| #31 | TS="Tisane" |
| #32 | TS="Herbal Teas" |
| #33 | #19 OR #20 OR #21 OR #22 OR #23 OR #24 OR #25 OR #26 OR #27 OR #28 OR #29 OR #30 OR #31 OR #32 OR #33 OR #34 OR #35 |
| #34 | #17 AND #33 |

**Search in Chinese: CNKI; Results: 20**

SU=(cha) AND SU=(duonangluanchaozonghezheng+polycystic ovarian syndrome+polycystic ovary syndrome+women with polycystic ovary syndrome+ duonangluanchaozonghezheng+duonangxingluanchaozonghezheng+duonangluanchaozonghezhen g+ duonangluanchaozonghezheng+duonangxingluanchao+PCOS)

**Search in Chinese: Wanfang Database; Results: 31**

Zhuti:(cha) * Zhuti:(duonangluanchaozonghezheng or polycystic ovarian syndrome or polycystic ovary syndrome or women with polycystic ovary syndrome or duonangluanchaozonghezheng or duonangluanchaozonghezheng or duonangluanchaozonghezheng or duonangluanchaozonghezheng or duonangxingluanchao or PCOS)

**Search in Chinese: VIP database; Results: 0**

(M=cha)*(M=duonangluanchaozonghezheng+M=polycystic ovarian syndrome+M=polycystic ovary syndrome+M=women with polycystic ovary syndrome+M=duonangxingluanchaozonghezheng+M=duonangxingluanchaozonghezheng+M=du onangluanchaozonghezheng+M=duonangluanchaozonghezheng+M=duonangxingluanchao+M=P COS)

**Search in Chinese: CBM (SinoMed); Results: 12**

("cha"[bujiaquan:kuozhan] OR cha) AND ("duonangluanchaozonghezheng"[bujiaquan:kuozhan] OR duonangluanchaozonghezheng OR polycystic ovarian syndrome OR polycystic ovary syndrome OR women with polycystic ovary syndrome OR duonangxingluanchaozonghezheng OR duonangxingluanchaozonghezheng OR duonangluanchaozonghezheng OR duonangluanchaozonghezheng OR duonangxingluanchao OR PCOS)
