# Supplementary material for: Effects of Tea Consumption on Anthropometric Parameters, Metabolic Indexes and Hormone Levels of Women with Polycystic Ovarian Syndrome: A Systematic Review and Meta-Analysis of Randomized Controlled Trials
Source: Front Endocrinol (Lausanne). 2021 Dec 13;12:736867. doi: 10.3389/fendo.2021.736867 (PMC8710535; doi:10.3389/fendo.2021.736867)
Supplement: Supplementary file 2 [file DataSheet_2.pdf]

Supplementary Appendix 2

Study ID	Selection bias		Performance bias	Detection bias	Attrition bias	Reporting bias	Other bias
	Random sequence generation	Allocation concealment					
**Tehrani et al., 2017[14]**	Unclear risk (no randomization details provided)	Unclear risk (no randomization details provided)	Unclear risk (no blinding details provided)	Unclear risk (no blinding details provided)	Low risk (85.7% participants were included in the analysis)	Low risk (no potential sources of reporting bias)	Low risk (baseline balanced)
**Grant, 2009[24]**	Low risk (concealment of allocation was achieved via a computerized program)	Low risk (concealment of allocation was achieved via a computerized program)	Low risk (clinical outcome measurements were assessed by two reliable investigators who were blinded to allocation)	Low risk (clinical outcome measurements were assessed by two reliable investigators who were blinded to allocation)	Low risk (97.6% participants were included in the analysis)	Low risk (no potential sources of reporting bias)	unclear risk (insufficient information)
**Chan et al., 2016[25]**	Low risk (randomization was performed according to a randomization table with the allocation group sealed in opaque envelopes)	Low risk (randomization was performed according to a randomization table with the allocation group sealed in opaque envelopes)	Low risk (capsules were identical in appearance and both the participants and the investigators were blinded to which treatment group each participant was assigned to)	Low risk (capsules were identical in appearance and both the participants and the investigators were blinded to which treatment group each participant was assigned to)	Low risk (all participants were included in the analysis)	Low risk (no potential sources of reporting bias)	Low risk (baseline balanced)
**Husein et al., 2015[26]**	Low risk (participants were randomly assigned by RANDOM ALLOCATION software)	Low risk (participants were randomly assigned by RANDOM ALLOCATION software)	Low risk (no blinding details provided)	Low risk (no blinding details provided)	Low risk (89.3% participants were included in the analysis)	Low risk (no potential sources of reporting bias)	Low risk (baseline balanced)

**Mombaini et al., 2017**[27]	Unclear risk (no randomization details provided)	Unclear risk (no randomization details provided)	Low risk (the researchers were blinded to the identities of the subjects and which group they belong to)	Low risk (the researchers were blinded to the identities of the subjects and which group they belong to)	Low risk (90.0% participants were included in the analysis)	Low risk (no potential sources of reporting bias)	Low risk (baseline balanced)
**Farhadian et al., 2020**[28]	Low risk (randomization was performed by a person who did not know the research project)	Low risk (randomization was performed by a person who did not know the research project)	Low risk (double-blind and capsules were identical in appearance)	Low risk (double-blind and capsules were identical in appearance)	Low risk (all participants were included in the analysis)	Low risk (no potential sources of reporting bias)	Low risk (baseline balanced)
