# Supplementary material for: Effects of Tea Consumption on Anthropometric Parameters, Metabolic Indexes and Hormone Levels of Women with Polycystic Ovarian Syndrome: A Systematic Review and Meta-Analysis of Randomized Controlled Trials
Source: Front Endocrinol (Lausanne). 2021 Dec 13;12:736867. doi: 10.3389/fendo.2021.736867 (PMC8710535; doi:10.3389/fendo.2021.736867)

Supplementary Appendix 3

Study omitted	Estimate	[95% Conf.	Interval]
Chan (2006)	-.0032305	-.04806391	.04160292
Mombaini (2017)	-.01571189	-.0656345	.03421072
Farhadian (2020)	-.04000002	-.06583623	-.01416381
Combined	-.02042968	-.05876032	.01790097

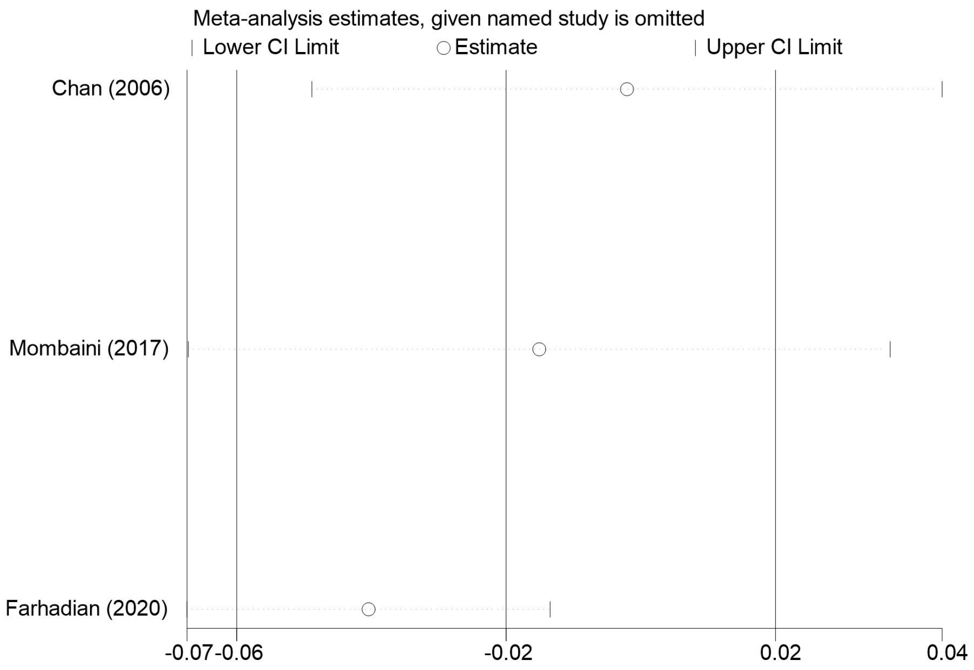

Supplement: Supplementary file 3 [file DataSheet_3.pdf]
