# Supplementary figures and images for: Effects of Tea Consumption on Anthropometric Parameters, Metabolic Indexes and Hormone Levels of Women with Polycystic Ovarian Syndrome: A Systematic Review and Meta-Analysis of Randomized Controlled Trials
Source: Front Endocrinol (Lausanne). 2021 Dec 13;12:736867. doi: 10.3389/fendo.2021.736867 (PMC8710535; doi:10.3389/fendo.2021.736867)

## Supplementary Appendix 4

| Study omitted | Estimate   | [95% Conf. Interval] |
|---------------|------------|----------------------|
| Chan (2006)   | -.52587289 | -6.9732785 5.9215326 |
| Grant (2009)  | -1.2575966 | -4.8725314 2.3573384 |
| Husein (2015) | .90894204  | -1.3420541 3.1599381 |
| Combined      | .24354953  | -2.3855006 2.8725997 |

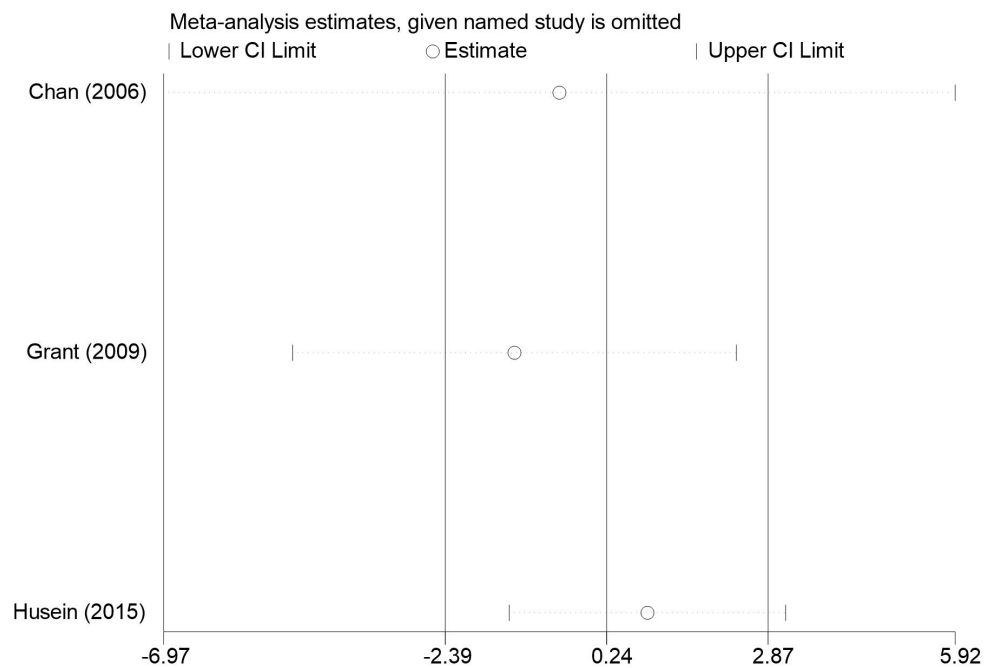

Supplement: Supplementary file 4 [file DataSheet_4.pdf]

Supplementary Appendix 5

| Study omitted  | Estimate   | [95% Conf. Interval]  |
|----------------|------------|-----------------------|
| Grant (2009)   | -.71999991 | -1.0894588 -.35054109 |
| Tehrani (2017) | -.18000001 | -.337832 -.02216799   |
| Combined       | -.42310314 | -.949661 .10345472    |

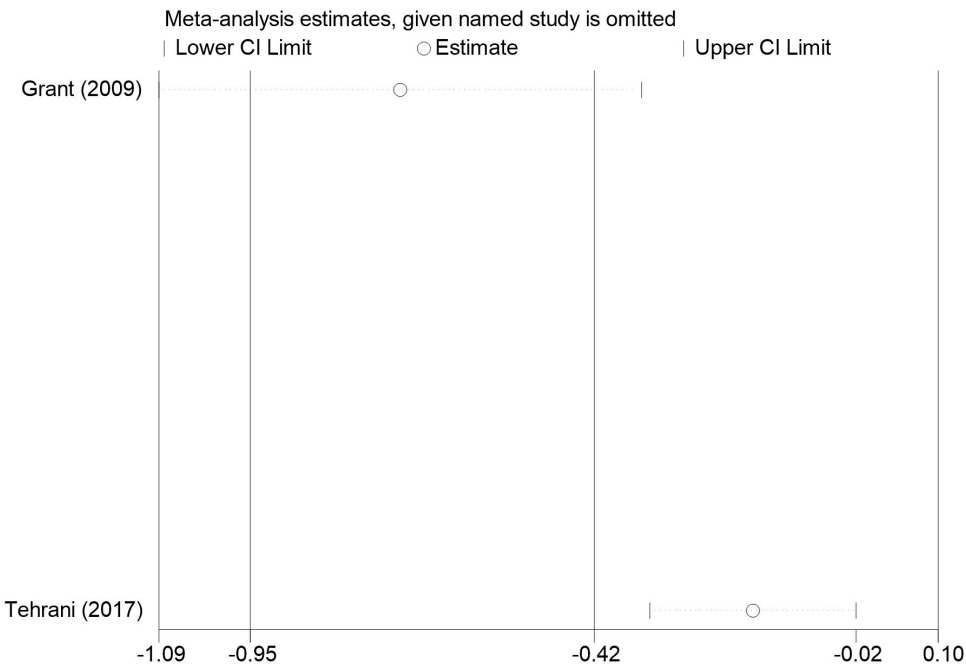

Supplement: Supplementary file 5 [file DataSheet_5.pdf]
